# The effect of medial calcar support on proximal humeral fractures treated with locking plates

**DOI:** 10.1186/s13018-022-03337-5

**Published:** 2022-10-28

**Authors:** Chun-Yu Hung, Chia-Yi Yeh, Po-Chong Wen, Wen-Ling Yeh, Shih-Jie Lin

**Affiliations:** 1 Department of Orthopaedic Surgery, Chang Gung Memorial Hospital, Yunlin, Taiwan; 2 Department of Orthopaedic Surgery, Jen-Ai Hospital, Taichung, Taiwan; 3 Department of Orthopaedic Surgery, Chang Gung Memorial Hospital, Linkou, Taiwan; 4 Department of Orthopaedic Surgery, Lotung Poh-Ai Hospital, Yilan, Taiwan; 5 Department of Orthopaedic Surgery, New Taipei Municiple TuCheng Hospital, Chang Gung Memorial Hospital, New Taipei City, Taiwan; 6 Department of Chemical Engineering, National Tsing Hua University, Hsinchu, Taiwan

**Keywords:** Proximal humeral fracture, Locking plate, Medial calcar support, Functional outcome

## Abstract

**Background:**

Studies have reported mixed results on the importance of medial calcar support for the treatment of proximal humeral fractures. The purpose of this study was to compare radiographic and functional outcomes of patients who had displaced proximal humeral fractures with varying levels of medial support.

**Methods:**

We performed a retrospective comparative cohort study. The study was conducted at a Level III trauma center in Taiwan. Seventy patients with proximal humeral fractures were collected retrospectively from 2015 to 2019. Only patients with two-, three-, or four-part types (Neer type I, II, or III) of displaced proximal humeral fractures were included in this study. However, patients with head-split fracture patterns, shoulder dislocation, prior shoulder trauma, and poor fracture reduction present in postoperative films were excluded. We assessed the radiographic outcomes, including the reduction score and amount of impaction in the humeral head. The functional outcome was evaluated based on the Constant score.

**Results:**

Patients were grouped into the intact medial calcar group and the medial calcar deficiency group. In a subgroup analysis, the group with intact medial support had a significantly lower amount of impaction and a higher Constant score compared with the medial calcar deficiency group. Additionally, the groups with intact medial support had a nonsignificant difference in the Constant score between the affected side and the contralateral side.

**Conclusion:**

The amount of impaction and the reduction score in the humeral head at the 12-month radiographic follow-up were significantly higher in the group with  medial support deficiency. However, the reduction score after surgery exhibited no difference. This implies that the inherent nature of medial comminution of proximal humeral fracture may lead to inferior radiographic outcomes.

## Introduction

Proximal humeral fractures are one of the most common shoulder injuries in the elderly population. Fractures of the proximal humerus account for approximately 45% of all humeral fractures and 10% of all fractures in patients older than 65 years [1].

Numerous treatment options can be applied to proximal humeral fractures. Open reduction and internal fixation (ORIF) with locking plates is one of the most common techniques for treating displaced proximal humeral fractures. In comparison with conventional fixation plates, the locking plate system provides rotational and angular stability and greater resistance to failure, especially in cases of osteoporosis [2]. Locking plates can also fix and stabilize bone fragments without causing screw–plate friction and thus, provide more stability in osteoporotic bone [3, 4]. However, use of these plates is associated with 36–49% of complications in elderly patients with poor bone quality and low blood supply [5, 6]. Common complications such as loss of reduction, screw perforation, and ischemic osteonecrosis of the humeral head have been reported [5, 7–9]. The three primary reasons for secondary displacement are poor bone quality, the stiffness of the implant, and high peak stress at the bone–implant interface [10]. These complications are more likely to occur in fractures with medial comminution. Several surgical techniques, including calcar screws, cement augmentation, fibular strut allograft, and bone grafting, have been used to increase the stability of locking plate fixation of proximal humeral fractures and thus, have improved clinical outcomes. The locking plate provides fixation strength because of the fixed-angle locking mechanism with diverging screws to occupy the volume of the humeral head [11].

The purpose of this study was to compare the clinical and radiographic outcomes of patients with proximal humeral fractures who received locking plate fixation with varying levels of medial support.

## Patients and methods

### Study design and setting

We reviewed the registry database of osteoporosis fractures at our institution, a Level III trauma center in Taiwan, after receiving approval from the Institutional Review Board of Chang Gung Memorial Hospital. From 2015 to 2019, 83 adult patients (> 18 years) with proximal humeral fractures were treated with Zimmer locking plates. Trauma fellowship training was compulsory for all operating surgeons. The study included 70 patients undergoing ORIF with the Zimmer plate.

### Participants

Patients with proximal humerus fractures were classified based on Neer’s [12] and Hertel’s [13] fracture classifications. Patients with acute fractures (< 14 days after injury) and displaced two-, three-, or four-part fractures were included in the study. Moreover, based on Bahrs’ criteria [14], only patients with good fracture reduction evident in postoperative images were included in our study. We excluded patients with inadequate postoperative reduction, a head-split fracture pattern, an associated shoulder dislocation, concomitant lesions, prior traumatic shoulder injury, or pathological fractures.

The risk factors of chronic kidney disease, chronic liver disease, and diabetes mellitus are irrelevant to fracture redisplacement after initial fracture reduction [15]. Therefore, we did not exclude patients with these diseases.

### Surgical techniques

We used the deltopectoral and deltoid-splitting approaches for proximal humeral fracture fixation. The plates were applied from the lateral to the bicipital groove and were fixed to the shaft with two or more cortical screws. To stabilize a fracture, at least six self-tapping 3.5-mm screws were inserted into the humeral head using an aiming device with fluoroscopic guidance. Fragments with greater or lesser tuberosity were fastened to the suture holes of the plate using sutures.

### Aftercare

A sling was used postoperatively for comfort. The patients were able to perform passively assisted range of motion activities immediately. Active resisted range of motion began 6 weeks postoperatively. Strengthening did not begin until 12 weeks postoperatively.

Our hospital’s standard of care for treating proximal humeral fractures includes outpatient follow-up at 2 weeks and at 1, 3, 6, 12, and 24 months. Clinical outcomes were assessed using the Constant–Murley scoring system (Constant) [16]. Standard true anteroposterior, Y, and axillary radiographs of the shoulder were obtained at regular follow-ups. A single surgeon (CYH) reviewed each radiograph, and the interobserver reliability was assessed by an independent reviewer (PCW). An independent surgeon (PCW) performed interval measurements twice at 3-week intervals.

### Outcome measurements

The head-shaft angulation was determined using the true anteroposterior view as described in other studies [17, 18].

The quality of fracture reduction was assessed using Bahrs’ criteria: 0 (perfect) = all three criteria ([A–C]) were met; 1 (good) = two of three criteria were met; 2 (fair) = one of three criteria was met; and 3 (poor) = none of the criteria were met. The criteria are as follows: (A) greater tuberosity with a side-to-side difference of < 5 mm, (B) no increased varus or valgus (± 15°) of the head fragment in the anteroposterior view, and (C) no increased retrotorsion or antetorsion (± 15°) of the head fragment in the axillary view [14]. The study only included fractures with a reduction score of 0 or 1 (perfect or good). A significant loss of reduction was defined as a deterioration of at least one of Bahrs’ three criteria.

**Fig. 1:**
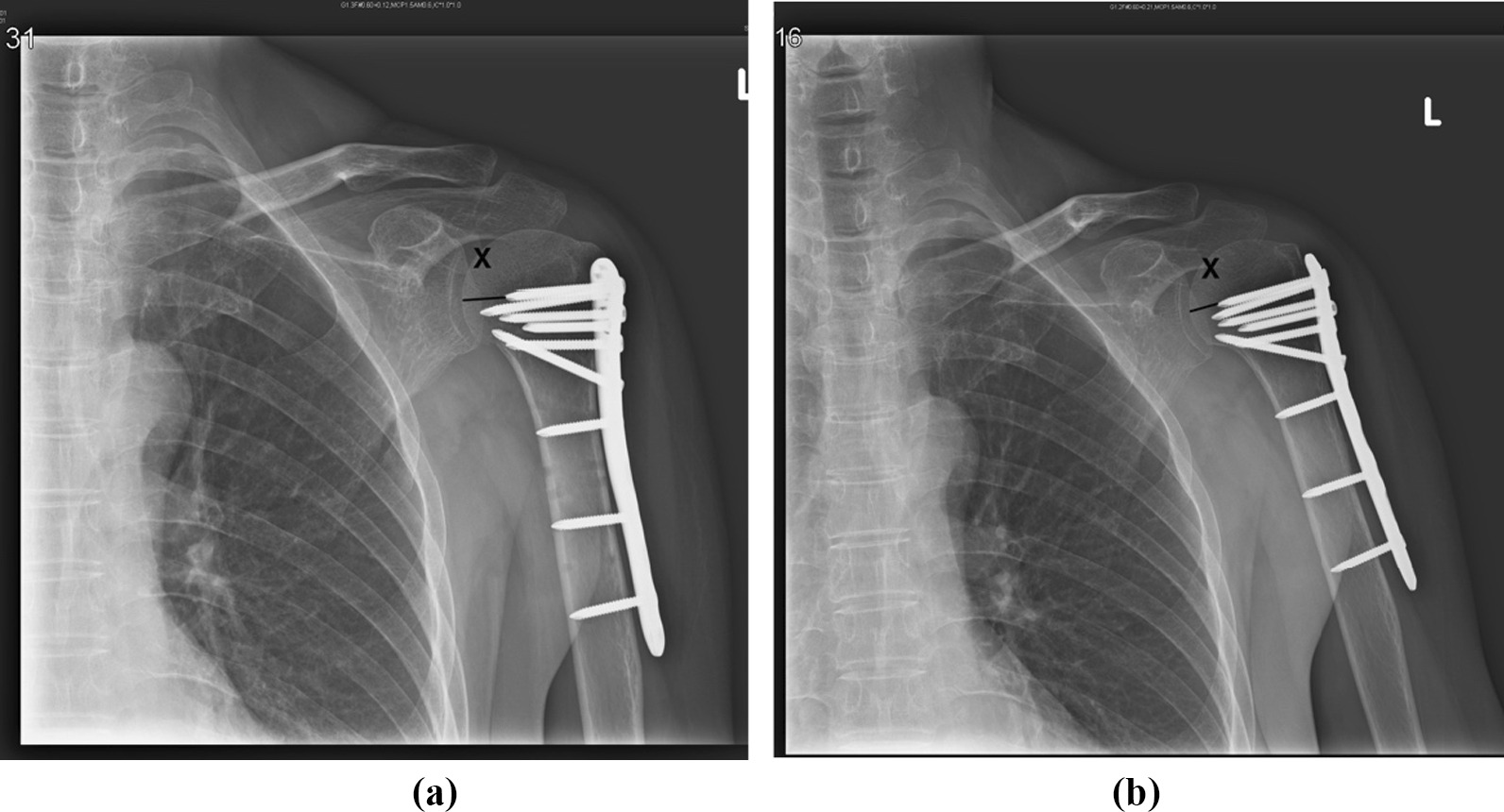
**a** The distance between the most cranial screw and humeral articular distance is measured in external rotation A-P view 1 day postoperatively (X = 13.51 mm). **b** 12 months postoperatively, the distance between the screw tip and articular surface is measured in the same method (X = 10.09 mm). The amount of head impaction is 13.51 mm–10.09 mm =  3.42 mm in this patient

We used a modified method proposed by Carbone et al. [19] to assess the amount of humeral head impaction (Fig. 1). The measurement of X (the distance) using Carbone’s method yields the true distance between the tip of the most cranial screws and the humeral articular surface of the head. The differences between X in the baseline postoperative plain films and at the 12-month follow-up were used to determine the extent of impaction. A functional outcome was evaluated on the basis of the Constant function score at the 12-month follow-up.

### Medial calcar support

Medial calcar support consists of two parts, namely the length of the dorsomedial metaphyseal extension and the integrity of the medial hinge, and these are also the most important predictors of fracture-induced humeral head ischemia [13]. The length of the dorsomedial metaphyseal extension means the length of the metaphyseal head extension and is classified as < 8 mm (calcar disruption) or ≥ 8 mm (intact calcar). The integrity of the medial hinge was calculated as the head dislocation attached to the diaphysis: > 2 mm (hinge disruption) or ≤ 2 mm (intact hinge). Medial calcar support deficiency is defined as either calcar disruption, hinge disruption, or both.

### Statistical analysis

All data analyses were conducted using SPSS 17.0 (SPSS Inc., Chicago, IL, USA). Descriptive statistics were used to describe the characteristics of the data. Results of different fracture types were compared using Fisher’s exact test for proportions and Student’s *t* test for average values. The nominal and ordinal variables are summarized as percentages. Fleiss’ generalized kappa coefficient was used to assess interobserver and intraobserver reliability. Statistical significance was set at *P* ≤ 0.05.

## Results

The study group included 70 patients with proximal humeral fractures who were followed-up for at least 1 year (average, 13.5 months; range, 12–24 months). Patients were aged 51 to 80 years (average, 64.2 years) with a male-to-female ratio of 19:51. The average operating time was 67 min (range, 52–85 min), and the average blood loss was 235 mL (range, 200–350 mL). The mean length of hospital stay was 5.5 days (range, 4–7 days). All patients were treated with Zimmer locking plates that had six screws, which included calcar screws, in the humeral head.

In a subgroup analysis, the extent of impaction was determined on the basis of medial calcar continuity (Table 1). We found no difference in postoperative reduction quality between the groups with medial support deficiency and those with intact medial support. However, the extent of impaction was significantly higher in patients with medial support deficiency. At the 12-month follow-up, we also observed that these patients had a significantly worse reduction score.

**Table 1:** Comparison of two levels of medial support

	Intact medial support (*N* = 37)	Medial support deficiency (*N* = 33)	*p*
Number of screws in head	6.0	6.0	1.000
Reduction score^#^ Postoperatively	0.5	0.6	0.444
Reduction score^#^ at l2-month follow-up	0.8	1.5	< 0.001*
Mean length of screw^a^, mm	39.8	39.8	0.913
Postoperative distance^b^, mm	4.1	4.2	0.598
Amount of impaction, mm	1.9	2.9	< 0.001*

a = Measured length of the most cranial screw on anteroposterior view

b = Actual distance between the screw tip and the articular surface (i.e., X)

^#^ = Bahrs’ criteria: 0 to 3

^*^ = difference is statistically significant, *P* ≤ 0.05

At the 12-month follow-up, the functional outcome of the affected side was significantly different between the two groups (intact medial support group and medial support deficiency group). At the 12-month follow-up, the Constant score of the affected side was lower than that of the contralateral side in both groups (Table 2). The difference between the affected side and the contralateral side was nonsignificant in the group with intact medial support. The Constant score differed significantly between the affected side and the contralateral side in the medial support deficiency group (*p *= 0.023).

**Table 2:** Constant scores of the two groups

Score	Intact medial support		Medial support deficiency	
	Affected	Contralateral	Affected	Contralateral
Constant Score	72.8 (60–85)	85.8 (78–92)	52.8 (46–72)	80.5 (71–87)

## Discussion

The optimal management of proximal humeral fractures remains controversial. Nonoperative treatment is reportedly effective in treating nondisplaced or minimally displaced fractures, but it has been shown to be ineffective in more complex fractures [20]. Although precontoured locking plates provide favorable results in many fracture treatment cases, treating complex fractures remains a challenge even with the use of this plate fixation device. Several studies have demonstrated high complication rates following locking plate osteosynthesis of proximal humeral fractures [21], [22]. The goal of our study was to evaluate the effect of medial calcar integrity on the radiographic and clinical outcomes of ORIF for proximal humeral fractures.

Despite the benefits of locking plate design and the update of biomechanical concepts such as the use of medial calcar screw, complication rates remain high after receiving locking plate fixation [23]. The most common complications related to plate fixation were osteonecrosis (4–33%), intra-articular screw perforation (5–20%), loss of fixation (3–16%), infection (4–19%), and impingement (7–11%) [23]. Previous studies have demonstrated that anatomic reduction and restoration of medial calcar support can reduce the risks of screw penetration, articular surface collapse, and osteonecrosis [24, 25]. Regarding our medical team, all our surgeons did their best to restore medial calcar support and used calcar screws routinely under direct vision. A biomechanical study demonstrated that inserting more than one calcar screw achieved no additional torsional or axial stability [26]. Additionally, a rotator cuff was sutured to the plate holes in all cases.

The successful management of proximal humeral fractures with an unstable medial column by using locking plate fixation depends on the restoration of a stable medial column, which provides stability, improves the rate of successful healing, and reduces complications [27, 28]. A biomechanical study demonstrated that medial comminution reduced the mean load to failure by 48% and the mean energy to failure by 44% [29]. Moreover, Zhang et al. [30] observed a clinically significant increase in varus collapse for three-and four-part proximal humeral fractures when there was no medial screw support at the fracture sites. This collapse situation was not observed in treating two-part fractures, suggesting that medial column screw is less important for fractures with greater intrinsic stability [29, 30]. However, the placement of calcar screws is effective for enhancing fixation stability of locking plates. It reduces the risk of a varus collapse with subsequent screw perforation by counteracting the varus deforming forces acting on the humeral head, thus resulting in a significantly higher reposition stability after 6 and 12 months [24, 28].

In our previous work [15], we confirmed that fractures with disrupted medial calcar support are associated with unfavorable clinical and radiographic outcomes. Additionally, we also identified that medial calcar disruption is a significant risk factor for predicting the osteonecrosis of humeral head and redisplacement of fracture reduction following ORIF [31]. To further verify the effect of medial support on plate fixation stability, we modified the method proposed by Carbone et al. [19] to evaluate the extent of humeral head impaction in the status of osteoporosis combined with medial support deficiency. Carbone et al. compared patients’ complications after they had received Humerusblock device fixation and demonstrated that the metaphyseal comminution pattern was more susceptible to head impaction than other factors and that humeral head impaction negatively impacted Constant score [19]. Our study demonstrated that group with medial calcar support deficiency had a greater extent of humeral head impaction and a lower Constant score than did the group with intact medial calcar support (Tables 1 and 2). The inherent nature of medial comminutions has been demonstrated to be linked to inferior clinical and radiographic outcomes. In addition to medial calcar support, the plate design can be optimized by using diverging screws to increase the volume of the humeral head occupied by peripheral screws, which is considered another important factor for evaluating the biomechanical strength of plate fixation [11]. Moreover, a cadaver study reported that more screws in the humeral head significantly increased the number of cycles before screw perforation [32]. As such, we believe that inserting more and longer screws into the humeral head may improve fixation stability and reduce screw perforation. Some surgeons modify their surgical techniques to minimize the risk of screw penetration by placing the screws 10 to 15 mm away from the articular surface [33]; however, the inserted screws may also be away from the subchondral bone, reducing the number of screws that should be used and possibly increasing the risk of loss of reduction [32]. Instead of using calcar screws, other surgeons have also modified their techniques by performing minimally invasive surgery through two incisions. To produce better results, the indications for these modified techniques need to be investigated in different fracture types.

Locking plates have different plate configurations and screw lengths and trajectories, which affect potential biomechanical performance and can optimize fracture reduction [11]. McDonald et al. compared the geometry of proximal humerus locking plates from seven manufacturers [11]. They found that the locking plates all displayed a conical pattern of screw distribution and that the large volume of a partial cone shape improved the resistance to failure. Regardless of the length of screws, the Zimmer plates had the lowest volume of the humeral head occupied by the most peripheral screws. Additionally, the Zimmer plates featuring the smallest bone–screw interface often result in the loss of hardware fixation. Clinicians should be aware of these construct differences when using locking plates because of their unfavorable effect on the biomechanical strength of fixation.

The current study has several limitations, including a small sample size, a retrospective design, and the use of different surgical approaches that may yield different results regarding medial calcar restoration and functional performance. The influence of surgical approach on study findings was not considered in this study. Some studies have reported that a deltopectoral approach produces better functional outcomes than a deltoid-splitting approach [34]. Yet, other studies have not found any difference between surgical approaches [35]. A prospective, large-scale study with biomechanical analysis is required to provide more evidence of the efficacy of surgical approaches for fracture treatment.

## Conclusion

In our comparison of patients with proximal humeral fracture without initial medial support versus those with intact medial support, the former had lower Constant scores, higher levels of loss reduction, and a greater extent of impaction at 1-year follow-up, even when treated with a locking plate system. Therefore, our findings suggest that clinicians should inform patients of these differences, particularly those without initial calcar support.

## Data Availability

The data used to support the findings of this study are available from the corresponding author upon request.
